# Hope and Resilience Related to Fear of COVID-19 in Young People

**DOI:** 10.3390/ijerph19095004

**Published:** 2022-04-20

**Authors:** David J. Javier-Aliaga, Gluder Quispe, Dámaris Quinteros-Zuñiga, Cristian E. Adriano-Rengifo, Michael White

**Affiliations:** 1 Center for Advanced Research in Neurosciences (CIAN), Universidad Peruana Unión (UPeU), Lima 15464, Peru; 2 Rectory, Universidad Peruana Unión (UPeU), Lima 15464, Peru; gluder@upeu.edu.pe; 3 Professional School of Psychology, Faculty of Health Sciences, Universidad Peruana Unión (UPeU), Tarapoto 22201, Peru; damaris@upeu.edu.pe; 4 Department of Psychology, Faculty of Health Sciences, Universidad Peruana Unión (UPeU), Lima 15464, Peru; cristianadriano@upeu.edu.pe; 5 Professional School of Communication Sciences, Faculty of Human Sciences and Education, Universidad Peruana Unión (UPeU), Lima 15464, Peru; michaelwhite@upeu.edu.pe

**Keywords:** hope, resilience, fear, COVID-19, youth

## Abstract

In the face of the psychological crisis of fear caused by the COVID-19 pandemic, it is relevant to know the positive impact of hope and resilience during this context. The purpose of this study was to determine the correlation between hope and resilience with fear of COVID-19 in young people. The design was non-experimental, cross-sectional, and correlational. The sample consisted of 192 young people living in Metropolitan Lima, Peru. We used the Hope-Despair Questionnaire, the Resilience Scale, and the COVID-19 Fear Questionnaire. The results show that there is a significant correlation between hope, resilience, and fear of COVID-19 in young people. On the other hand, a significant difference was found in resilience according to gender. Likewise, it was found that the variables hope and resilience explain 81% (R^2^ adjusted) of the fear of COVID-19 (F test = 21.53; *p* < 0.01). Hope and resilience are protective factors that have a positive impact when facing the fear of COVID-19. Thus, policies, programs, and public health strategies related to positive mental health should be promoted, with emphasis on hope and resilience.

## 1. Introduction

On 11 March 2020, the World Health Organization declared COVID-19 to be a pandemic [1]. COVID-19, similar to previous outbreaks of coronaviruses such as SARS-CoV in 2002 [2] and MERS-CoV in 2012 [3], has been characterized as an endemic agent and a major public health threat [4]. This is primarily due to its easy transmission from person to person through droplets or direct contact [5,6,7,8]. By the end of 2021, Peru registered more than 200,000 deaths due to the COVID-19 crisis [9], which is one of the highest mortality rates in the world [10,11].

With the high infection rate and relatively high mortality rate, people naturally began to worry about COVID-19, as its rapid and invisible transmission [12], particularly by asymptomatic carriers [7,13], has generated fear in people, compromising their mental health. This fear can lead people to erratic behavior [14], which in complicated cases can manifest itself in a clinical picture of delirium [15]. Additionally, the restrictions imposed in an effort to control the spread of the pandemic can lead people to feel as if they will not be able to experience positive emotions and that their condition will not improve, which can lead to hopelessness [16]. There is also evidence that fear of COVID-19 is interconnected to a network of symptoms such as socioeconomic concerns, xenophobia, traumatic stress symptoms, compulsive checking, and reassurance seeking [17]. Several studies have shown multiple adverse effects of the COVID-19 pandemic on people’s mental health [18,19,20,21,22,23,24,25,26,27]. 

During the first days of the COVID-19 outbreak, a moderate to severe psychological impact of depression, anxiety, and stress symptoms was reported in the general population in China [28]. Studies in Spain reported that younger people showed elevated levels of depression, anxiety, and stress [29], as well as an emotional impact reflected in the fear of coronavirus infection, illness, and death [30]. Additionally, high school, undergraduate, and graduate students in Mexico presented a greater presence of anxiety and depression during confinement [31]. In Peru, a higher prevalence of anxiety was found in young university students at the beginning of the confinement [32]. In another study, adults showed greater fear of death due to coronavirus than young people [33]. However, the mean scores of young people are considerable and deserve to be studied. In Argentina, young people were found to have feelings of sadness, low mood, and increased alcohol consumption from the onset of the pandemic [34].

Regarding gender differences, several studies have reported that the COVID-19 pandemic has a greater impact on the mental health of women than men [19,20,24,27,35,36]. However, a study in Nigeria on experiences of psychological distress during the COVID-19 pandemic found no significant differences according to gender [37]. Longitudinal studies have shown that women had greater negative effects on their mental health than men, although the differences faded over time [21,38,39].

It is crucial that we do not ignore the psychological impact that this pandemic will have on individuals and society, which may even persist long after the pandemic has ended [40,41,42]. Faced with this situation, it is important to know the related variables or protective factors for preventing or reducing the negative effects of the COVID-19 pandemic on the mental health of young people.

Resilience has been defined as a person’s ability to respond effectively to trauma, adversity, or severe stress [43,44], with effective and active problem-solving and coping reflected in a resilient coping pattern [45]. Moreover, it is characterized by being dynamic, transforming and adapting throughout the subject’s life [46]. Resilience has a positive effect on the mental well-being, pain acceptance, and physical health of the individual [43,44,45,46,47]. It was found that resilience can promote adaptation and positive coping in people living with HIV and chronic pain [48]. It was also shown that it plays an important role as a protective variable in the quality of life at the level of mental health in patients with chronic renal failure [49]. In an international study, it was found that university students with high levels of resilience experienced lower levels of psychological disorders compared with students with low levels of resilience [50]. A recent study found that male students have higher levels of resilience than female students [51]. Resilience was also found to correlate inversely with anxiety, depression [52], and fear of COVID-19 [53]. In addition, a low level of depression is a predictor of resilience [48]. Likewise, resilience is a predictor and mediating variable of fear of COVID-19 [54,55]. Finally, resilience was found to be an important factor influencing levels of physical activity change during COVID-19 confinement [56]. It is evident that resilience represents an essential objective for psychological intervention in a public health emergency [27].

Hope is a driving force that energizes a person for future adaptation and finding meaning in life [57]. Initially, the study of hope in humans had a one-dimensional cognitive conceptualization based solely on achieving goals [58]. Subsequently, instruments emerged that postulated a multidimensional structure [59], although they did not consider the spiritual aspect of the human being. There is a more holistic theory that includes a dimension called “transcendent hope” that focuses on the belief in a future life, trusting in the promises of a god or higher power [60]. Hope includes a positive outlook on life even in the face of adversity and positively impacts a person’s mental health and well-being [60,61]. It plays an important role in recovery during hospital treatment [62]. Higher levels of hope have been found to reduce the suffering of cancer patients [63], including reducing the fear of death [57]. There is also evidence that women are more likely to experience a certain degree of hope compared with men [64]. Hope leads to the development of preventive behaviors that decrease the risk of disease [65]. Recently, the protective role of hope against the negative impact of fear of COVID-19 was reported [66].

Although evidence suggests that resilience and hope can have a positive impact on the mental health of people suffering from fear, anxiety, or severe stress, it is unclear whether these can act as protective factors in preserving one’s mental well-being in the midst of a global health emergency. Therefore, the main objective of this study was to identify and explore the correlation between hope and resilience with fear of COVID-19 in young college students.

## 2. Materials and Methods

### 2.1. Study Design and Participants

This was a non-experimental, cross-sectional, correlational study. The sample consisted of 192 young people residing in Metropolitan Lima (Peru), selected by non-probabilistic purposive sampling. Young men and women, between 18 and 29 years old, who were baptized members of a religious community were included. Participants who had been diagnosed with COVID-19 were excluded. Table 1 presents the sociodemographic characteristics of the participants, of whom 79.2% (152) were female and 18 to 29 years old. Regarding marital status, 93.2% (179) were single, and 6.8% (13) were married. Regarding religiosity, 83.9% (161) were baptized more than 6 years ago in the Seventh-day Adventist Church. Regarding place of origin, most of the respondents were from the coast (51%). Regarding educational level, the young people said they were university students and had not been diagnosed with COVID-19. Finally, it is evident that there was a statistically significant difference in marital status according to gender.

### 2.2. Instruments Used for Data Collection

The Test of Hope-Despair (Test de Esperanza-Desesperanza; TED-R) was developed by Pereyra in 2013 [60]. In this study, sub-scale E, composed of eight items, has been considered. The type of response of the items are: always, many times, “sometimes yes, sometimes no”, very rarely, and never. Example of scale questions: “I look towards tomorrow placing my trust in God” and “I believe in God’s promise that there is a happy world beyond earthly life”. For this study, the reliability of the TED-R was 0.775, according to Cronbach’s alpha.

The Resilience Questionnaire (BRCS) by Sinclair and Wallston [67] is composed of four items in a single dimension. The item responses are Likert-type (Never (nunca), Rarely (muy pocas veces), Sometimes (a veces), Frequently (muchas veces), and Always (siempre)), with scores ranging from 4 to 20. As an example, the first item on the scale is: “I look for creative ways to change difficult situations” (“Busco formas creativas para cambiar las situaciones difíciles”). In this study, the version translated by Limonero et al. (2014) was used. In this study, the reliability of the BRCS was 0.703, according to Cronbach’s alpha.

The Fear of COVID-19 scale (FC-19S) by Ahorsu et al. [12] presents seven items with a Likert-type scale. Responses include “strongly disagree”, “disagree”, “neither agree nor disagree”, “agree”, and “strongly agree”. This is a unidimensional scale. The minimum possible score for each question is 1, and the maximum is 5. The total score is calculated by adding the score for each item (ranging from 7 to 35). Given the time frame of the study, a validated Spanish version of the Fear of COVID-19 scale was not available; therefore, an ad hoc translation was made. For this study, the reliability of the FC-19S was 0.738, according to Cronbach’s alpha.

### 2.3. Procedure

Data collection was carried out during the first wave of the pandemic, specifically, between June and July 2020. As of 31 July 2020, Peru had 422,183 confirmed cases of COVID-19 with 19,408 deaths and a case fatality rate of 4.60% [68].

For data collection, a virtual form was used, created through Google Forms. The first part contained an informed consent form, which was presented with a question to receive the participant’s acceptance, the second part asked questions about sociodemographic information, and the third part contained the questionnaires for the study variables. The virtual form was sent to a WhatsApp group made up of young Christians located in Metropolitan Lima. The young people answered the form in Spanish. They were assured that their participation was voluntary and anonymous. Finally, the study was conducted in accordance with the Declaration of Helsinki and received the approval of the ethics committee of the Faculty of Health Sciences of the Universidad Peruana Unión.

### 2.4. Statistical Analysis

The SPSS version 25 statistical software was used for data processing and analysis. The descriptive analysis was performed using the mean, median, and standard deviation. In addition, the sociodemographic variables were described using frequencies and percentages, and the difference between groups was performed using Pearson’s chi-square statistical test and Student’s *t*-test. Correlation analysis was performed using Pearson’s correlation coefficient. Likewise, the minimum sample size required for the multivariate regression analysis was 107, according to G. Power 3.1.9.7 and taking into account the following parameters: effect size f2 = 0.15, α error prob = 0.05, power (1 − β error prob) = 0.95, and number of predictors = 2. Additionally, multivariate analysis was performed using multiple linear regression. Finally, SPSS PROCESS 4.0 macro model 4 was used to identify the effect of hope and resilience on fear of COVID-19 and the potential mediating role of resilience in this effect. A significance level of 5% was used for the analysis.

## 3. Results

This section presents the comparative, correlational, explanatory, and exploratory analysis of mediation and direct and indirect effects between the study variables. Table 2 shows the results of the descriptive-comparative analysis, where it is evident that there is no statistically significant difference in the variables hope and fear of COVID-19 according to gender. However, there is a difference in resilience according to gender.

Table 3 reveals that there is a statistically significant correlation between the variables hope, resilience, and fear of COVID-19 in college students, including a negative correlation between hope and fear of COVID-19 (*r* = −0.385; *p* < 0.01), a negative correlation between resilience and fear of COVID-19 (*r* = −0.394; *p* < 0.01), and a positive correlation between hope and resilience (*r* = 0.637; *p* < 0.01).

Table 4 shows that the equation of the multiple linear regression model is significant (F-test = 21.53; *p* < 0.01). Likewise, it is revealed that the variables hope and resilience explain 18% (R^2^ Adjusted) of the fear of COVID-19. In addition, the standardized beta weights indicate that resilience explains slightly better than hope in the model.

Finally, an exploratory analysis of mediation and the direct and indirect effects between the study variables was carried out, where fear of COVID-19 was considered as the dependent variable (Y), resilience as the mediating variable (M), and hope as the independent variable (X). The main results are as follows: first, the test(s) of X by M inter-action showed the following results: F = 0.108, df1 = 1.000, df2= 188.00, and *p* > 0.742, indicating that it is not feasible to pursue a moderation analysis. Second, when including the indirect effects of hope (X) on COVID-19 fear (Y) mediated through resilience (M), the results were: β = −0.159; *t* = −2.63; *p* < 0.001. Finally, the results of the direct and indirect effect as well as the bootstrap confidence interval (the absence of 0 in the interval) suggest the mediating role of resilience (Table 5). Additionally, the Sobel test showed that the partial mediation effect described in the model was statistically significant (*p* < 0.001). Number of bootstrap samples for percentile bootstrap confidence intervals: 50,000.

## 4. Discussion

The studies that have been undertaken in the context of the COVID-19 pandemic reveal that a prolonged and increasing exposure to risk factors deteriorates mental health, quality of life, and its perception [69]. Peruvian university students are no exception, and they have also had to adapt their academic routine to the virtual or remote modality, generating adverse emotional repercussions such as stress, anxiety, and fear of infection [70]. Given this situation, it is relevant to analyze the psychological factors associated with effective emotional management and coping with these challenges; therefore, the aim of this research study was to analyze whether hope and resilience predict fear of COVID-19 in young Peruvian university students.

Although no significant differences were found with respect to the level of hope and fear of COVID-19 depending on the gender of the participants, a difference was found with respect to the level of resilience, in favor of males. Evidently, this group has a greater capacity to face problems creatively, exercise situational control, and overcome losses. This could in part be related to the processes of socialization and learned roles, as well as in the experiences and sociocultural gender attributions that emerged around the pandemic. This opens a space for future studies, including considerations for post-pandemic psychosocial intervention. Other studies referring to these variables, such as one carried out in 420 Peruvian university students, showed that women and older people were more likely to experience hope [64]. In addition, there is a report on 51 Mexican university students that also identified a higher level of resilience in males [51]. Regarding fear related to COVID-19, studies conducted in Turkey, Italy, the Philippines, and Bangladesh in university students identified that female groups showed higher levels of fear, even though mortality rates have been higher among males who contracted the virus [71,72,73]. In addition, students with higher levels of fear also had higher levels of anxiety and lower sleep quality; there is a greater tendency among female students to show greater negative affectivity, although there are mediating factors that may intervene in its manifestation, such as age or culture [74].

The largest effect size was found in the correlation between hope and resilience. Evidently, a greater capacity to overcome crises and defeats, as well as the ability of the university students who were evaluated to overcome the state of extreme distress and stress, is associated with higher levels of hope in terms of perseverance and confidence in the future. The relationship between COVID-19-related fear and the hope and resilience constructs was inverse and moderate to weak. Thus, in the group of students evaluated, a lower level of hope is linked to a greater experience of fear of becoming infected with COVID-19 or even of losing one’s life to this disease. These findings are consistent with a study in India with 412 people, where it was observed that fear of COVID-19 negatively affected mental health through rumination; however, it was identified that hope can play a mediating role in coping with difficulties and overcoming adversities, as they have a greater capacity for resilience to the indirect negative impact of fear of COVID-19 [66,75]. Additionally, in Belgium and the Netherlands, it was found that fear of COVID-19 correlated with higher levels of depression, stress, and anxiety. However, in participants with high scores for mindfulness, optimism, and resilience this relationship was attenuated. Clearly, optimism, mindfulness, and resilience may serve as protective components against the negative mental health symptoms associated with COVID-19 fear and distress [76].

Regarding hope as a predictor of fear of COVID-19, it is inferred that feeling hopeful implies the perception that peace of mind and well-being are attainable; therefore, these conditions can generate a reduction in the uncertainty and tensions inherent to fear and dread. This interpretation coincides with a study that included 929 adults who were diagnosed by COVID-19, where it was identified that hope, among other variables, significantly predicted resilience, while intolerance to uncertainty and fear COVID-19 significantly impacted resilience [55].

Resilience was also identified as a predictor of fear of COVID-19. Evidently, this ability to face extreme situations, with flexibility and willingness to overcome, allows students to be strengthened and to have a greater experience in facing the threats and uncertainties that fear causes. This finding converges with another report from China, in a study with 1208 people, where it was identified that fear of traveling, as one of the consequences left by the pandemic, was significantly linked to different coping strategies, which increased people’s psychological resilience and the adoption of cautious behaviors [77].

### Limitations and Future Studies

Among the limitations of this study are the small size of the sample and the non-randomization in the incorporation of the participants, which does not allow for a generalization of the results beyond the age group studied. Likewise, the voluntary and virtual participation of the students could imply bias related to motivation or access to the technological means necessary to access the data collection form. Finally, the majority composition of the sample by women and members of a religious community could lead to a selection bias.

The results of this study show the positive impact of hope and resilience when facing the fear of COVID-19 in young people. However, the impact of these variables is likely to vary according to the specific conditions of each population. Future studies could include other variables related to the psychological impact of the pandemic, such as anxiety, post-traumatic stress, grief, pandemic fatigue, and spiritual care, among others, considering the importance of gender as an intervening variable [33,78].

Furthermore, while the cross-sectional design of the study has made it possible to analyze the dynamics between the variables at a given time, future studies should investigate the phenomenon from a longitudinal perspective—i.e., at different stages of the COVID-19 pandemic. In addition, it is suggested to carry out research considering moderation and/or mediation of hope and resilience on fear.

## 5. Conclusions

It is concluded that resilience was higher in the male university students evaluated, that hope and resilience were found to be inversely and significantly related to fear of COVID-19, and that the variables of hope and resilience were positively correlated with each other with the highest effect size found in the study. Regarding the predictive analysis, it was identified that both hope and resilience predicted fear related to COVID-19, showing the relevance of controlling these factors for maintaining emotional balance in the pandemic situation.

## Figures and Tables

**Table 1:** Sociodemographic analysis of the sample (*n* = 192).

Variables	Total (*n* = 192)		Gender				*p*
			Female (*n* = 152)		Male (*n* = 40)		
	*n*	%	*n*	%	*n*	%	
Place of origin							0.660
Coast	98	51.0	80	52.6	18	45.0	
Mountains	66	34.4	50	32.9	16	40.0	
Jungle	28	14.6	22	14.5	6	15.0	
Marital status							0.020 *
Single	179	93.2	145	95.4	34	85.0	
Married	13	6.8	7	4.6	6	15.0	
Years of baptism in the Christian church							0.668
Less than 5 years ago	31	16.1	25	16.4	6	15.0	
Between 6 and 10 years ago	67	34.9	50	32.9	17	42.5	
Between 11 and 20 years ago	86	44.8	71	46.7	15	37.5	
Over 21 years ago	8	4.2	6	3.9	2	5.0	

Pearson’s chi-squared test was used. * *p* < 0.05.

**Table 2:** Descriptive analysis of hope, resilience, and fear of COVID-19.

	Total Sample (*n* = 192)			Female (*n* = 152)			Male (*n* = 40)			*p*
	M	Mdn	SD	M	Mdn	SD	M	Mdn	SD	
Hope	27.05	28.00	2.99	26.87	28.00	2.94	27.75	27.00	3.11	0.112
Resilience	15.97	16.00	2.01	15.78	16.00	1.90	16.68	16.50	2.27	0.026 *
Fear of COVID-19	11.48	11.00	2.97	11.59	11.00	2.93	11.08	11.00	3.12	0.335

M = Mean; Mdn = Median; SD = Standard Deviation. Student’s *t*-test was used. * *p* < 0.05.

**Table 3:** Correlation between hope, resilience, and fear of COVID-19.

	Hope	Resilience	Fear of COVID-19
Hope	1		
Resilience	0.637 **	1	
Fear of COVID-19	−0.385 **	−0.394 **	1

** *p* < 0.01.

**Table 4:** Results of the multiple linear regression analysis.

	R	R^2^	R^2^ Adjusted	F	*p*	B	Beta	*t*	*p*	TOL	VIF
Constant	0.43	0.19	0.18	21.53	0.000	23.44	-	12.62	0.000 ***		
Hope						−0.223	−0.225	−2.64	0.009 *	0.594	1.685
Resilience						−0.372	−0.251	−2.95	0.004 *	0.594	1.685

* *p* < 0.05; *** *p* < 0.001. TOL = Tolerance; VIF = Variance Inflation Factor.

**Table 5:** Exploratory analysis of total, direct, and indirect effect.

The Indirect Effect of the Mediating Role of Resilience	Total Effect	Direct Effect	Indirect Effect	Bootstrap Confidence Interval—Standardized (BoLLCI-BoULCI)
Hope → Fear of COVID-19	−0.382	−0.223	−0.159	−0.265–−0.053

LLCI and ULCI: lower and upper levels of confidence interval.

## Data Availability

All data accumulated in the study were available to the authors. Data are not published openly due to privacy issues, but analyzed data are available from the authors upon request.

## References

[1] WHO Director-General’s Opening Remarks at the Media Briefing on COVID-19—11 March 2020. https://www.who.int/director-general/speeches/detail/who-director-general-s-opening-remarks-at-the-media-briefing-on-covid-19---11-march-2020.

[2] Zhong N.S., Zheng B.J., Li Y.M., Poon L.L.M., Xie Z.H., Chan K.H., Li P.H., Tan S.Y., Chang Q., Xie J.P. (2003). Epidemiology and cause of severe acute respiratory syndrome (SARS) in Guangdong, People’s Republic of China, in February, 2003. Lancet.

[3] Wang N., Shi X., Jiang L., Zhang S., Wang D., Tong P., Guo D., Fu L., Liu X., Arledge K.C. (2013). Structure of MERS-CoV spike receptor-binding domain complexed with human receptor DPP4. Cell Res..

[4] Rothan H.A., Byrareddy S.N. (2020). The epidemiology and pathogenesis of coronavirus disease (COVID-19) outbreak. J. Autoimmun..

[5] Li Q., Guan X., Wu P., Wang X., Zhou L., Tong Y., Ren R., Leung K.S.M., Lau E.H.Y., Wong J.Y. (2020). Early transmission dynamics in Wuhan, China, of novel coronavirus-infected pneumonia. N. Engl. J. Med..

[6] Wang D., Hu B., Hu C., Zhu F., Liu X., Zhang J., Wang B., Xiang H., Cheng Z., Xiong Y. (2020). Clinical Characteristics of 138 Hospitalized Patients with 2019 Novel Coronavirus-Infected Pneumonia in Wuhan, China. JAMA-J. Am. Med. Assoc..

[7] Rothe C., Schunk M., Sothmann P., Bretzel G., Froeschl G., Wallrauch C., Zimmer T., Thiel V., Janke C., Guggemos W. (2020). Transmission of 2019-NCOV infection from an asymptomatic contact in Germany. N. Engl. J. Med..

[8] De Chang M., Lin M., Wei L., Xie L., Zhu G., Cruz C.S.D., Sharma L. (2020). Epidemiologic and Clinical Characteristics of Novel Coronavirus Infections Involving 13 Patients Outside Wuhan, China. JAMA-J. Am. Med. Assoc..

[9] Plataforma Digital Única del Estado Peruano (2021). Coronavirus (COVID-19) en Perú. https://www.gob.pe/coronavirus.

[10] Johns Hopkins University Medicine (2021). COVID-19 Dashboard. https://coronavirus.jhu.edu/map.html.

[11] OMS (2021). COVID-19 Dashboard. https://covid19.who.int/.

[12] Ahorsu D.K., Lin C.-Y., Imani V., Saffari M., Griffiths M.D., Pakpour A.H. (2020). The Fear of COVID-19 Scale: Development and Initial Validation. Int. J. Ment. Health Addict..

[13] Wu Z., McGoogan J.M. (2020). Characteristics of and Important Lessons from the Coronavirus Disease 2019 (COVID-19) Outbreak in China: Summary of a Report of 72,314 Cases from the Chinese Center for Disease Control and Prevention. JAMA J. Am. Med. Assoc..

[14] Ho C.S., Chee C.Y., Ho R.C. (2020). Mental Health Strategies to Combat the Psychological Impact of COVID-19 beyond Paranoia and Panic. Ann. Acad. Med. Singap..

[15] Sher Y., Rabkin B., Maldonado J.R., Mohabir P. (2020). A Case Report of COVID-19 Associated Hyperactive ICU Delirium with Proposed Pathophysiology and Treatment. Psychosomatics.

[16] American Psychological Association (2020). APA Dictionary of Psychology. https://dictionary.apa.org/hopelessness.

[17] Taylor S., Landry C.A., Paluszek M.M., Fergus T.A., McKay D., Asmundson G.J.G. (2020). COVID stress syndrome: Concept, structure, and correlates. Depress. Anxiety.

[18] Planchuelo-Gómez Á., Odriozola-González P., Irurtia M.J., de Luis-García R. (2020). Longitudinal evaluation of the psychological impact of the COVID-19 crisis in Spain. J. Affect. Disord..

[19] Hawes M.T., Szenczy A.K., Klein D.N., Hajcak G., Nelson B.D. (2021). Increases in Depression and Anxiety Symptoms in Adolescents and Young Adults during the COVID-19 Pandemic. Psychol. Med..

[20] Ripoll J., Contreras-Martos S., Esteva M., Soler A., Serrano-Ripoll M.J. (2021). Mental health and psychological wellbeing during the COVID-19 lockdown: A longitudinal study in the balearic islands (Spain). J. Clin. Med..

[21] González-Sanguino C., Ausín B., Castellanos M.A., Saiz J., Muñoz M. (2021). Mental health consequences of the COVID-19 outbreak in Spain. A longitudinal study of the alarm situation and return to the new normality. Prog. Neuro-Psychopharmacol. Biol. Psychiatry.

[22] Boden M., Zimmerman L., Azevedo K.J., Ruzek J.I., Gala S., Magid H.S.A., Cohen N., Walser R., Mahtani N.D., Hoggatt K.J. (2021). Addressing the mental health impact of COVID-19 through population health. Clin. Psychol. Rev..

[23] Cuadrado E., Arenas A., Moyano M., Tabernero C. (2021). Differential impact of stay-at-home orders on mental health in adults who are homeschooling or “childless at home” in time of COVID-19. Fam. Process.

[24] Pieh C., Budimir S., Humer E., Probst T. (2021). Comparing Mental Health during the COVID-19 Lockdown and 6 Months after the Lockdown in Austria: A Longitudinal Study. Front. Psychiatry.

[25] Pierce M., McManus S., Hope H., Hotopf M., Ford T., Hatch S.L., John A., Kontopantelis E., Webb R.T., Wessely S. (2021). Mental health responses to the COVID-19 pandemic: A latent class trajectory analysis using longitudinal UK data. Lancet Psychiatry.

[26] Kimhi S., Marciano H., Eshel Y., Adini B. (2020). Community and national resilience and their predictors in face of terror. Int. J. Disaster Risk Reduct..

[27] Ran L., Wang W., Ai M., Kong Y., Chen J., Kuang L. (2020). Psychological resilience, depression, anxiety, and somatization symptoms in response to COVID-19: A study of the general population in China at the peak of its epidemic. Soc. Sci. Med..

[28] Wang C., Pan R., Wan X., Tan Y., Xu L., Ho C.S., Ho R.C. (2020). Immediate psychological responses and associated factors during the initial stage of the 2019 coronavirus disease (COVID-19) epidemic among the general population in China. Int. J. Environ. Res. Public Health.

[29] Ozamiz-Etxebarria N., Dosil-Santamaria M., Picaza-Gorrochategui M., Idoiaga-Mondragon N. (2020). Stress, anxiety, and depression levels in the initial stage of the COVID-19 outbreak in a population sample in the northern Spain. Cad. Saude Publica.

[30] Sandín B., Valiente R.M., García-Escalera J., Chorot P. (2020). Psychological impact of the COVID-19 pandemic: Negative and positive effects in Spanish people during the mandatory national quarantine. Rev. Psicopatol. Psicol. Clin..

[31] Camacho-Zuñiga C., Pego L., Escamilla J., Hosseini S. (2021). The impact of the COVID-19 pandemic on students’ feelings at high school, undergraduate, and postgraduate levels. Heliyon.

[32] Apaza C., Seminario R., Santa-Cruz J. (2020). Factores psicosociales durante el confinamiento por el COVID-19—Perú. Rev. Venez. Gerenc..

[33] Becerra B.D., Becerra D. (2019). Ansiedad ante la muerte en adultos peruanos, durante la pandemia de la COVID-19. Rev. Cubana Enferm..

[34] Alomo M., Gagliardi G., Peloche S., Somers E., Alzina P., Prokopez C.R. (2020). Efectos psicológicos de la pandemia COVID-19 en la población general de Argentina. Rev. Fac. Cienc. Med. Cordoba.

[35] Ausín B., González-Sanguino C., Castellanos M.Á., Muñoz M. (2021). Gender-related differences in the psychological impact of confinement as a consequence of COVID-19 in Spain. J. Gend. Stud..

[36] Del Río-Casanova L., Sánchez-Martín M., García-Dantas A., González-Vázquez A., Justo A. (2021). Psychological responses according to gender during the early stage of COVID-19 in Spain. Int. J. Environ. Res. Public Health.

[37] Olaseni A.O., Akinsola O.S., Agberotimi S.F., Oguntayo R. (2020). Psychological distress experiences of Nigerians during COVID-19 pandemic; the gender difference. Soc. Sci. Humanit. Open.

[38] Salfi F., Lauriola M., Amicucci G., Corigliano D., Viselli L., Tempesta D., Ferrara M. (2020). Gender-related time course of sleep disturbances and psychological symptoms during the COVID-19 lockdown: A longitudinal study on the Italian population. Neurobiol. Stress.

[39] Fenollar-Cortés J., Jiménez Ó., Ruiz-García A., Resurrección D.M. (2021). Gender Differences in Psychological Impact of the Confinement During the COVID-19 Outbreak in Spain: A Longitudinal Study. Front. Psychol..

[40] Chan A.O.M., Chan Y.H. (2004). Psychological impact of the 2003 severe acute respiratory syndrome outbreak on health care workers in a medium size regional general hospital in Singapore. Occup. Med..

[41] Hawryluck L., Gold W.L., Robinson S., Pogorski S., Galea S., Styra R. (2004). SARS control and psychological effects of quarantine, Toronto, Canada. Emerg. Infect. Dis..

[42] Van Bortel T., Basnayake A., Wurie F., Jambai M., Koroma A.S., Muana A.T., Hann K., Eaton J., Martin S., Nellums L.B. (2016). Psychosocial effects of an Ebola outbreak at individual, community and international levels. Bull. World Health Organ..

[43] Sturgeon J.A., Zautra A.J. (2010). Resilience: A new paradigm for adaptation to chronic pain. Curr. Pain Headache Rep..

[44] Bartley E.J., LaGattuta N.R., Robinson M.E., Fillingim R.B. (2019). Optimizing resilience in orofacial pain: A randomized controlled pilot study on hope. Pain Rep..

[45] Sinclair V.G., Wallston K.A. (2004). The development and psychometric evaluation of the Brief Resilient Coping Scale. Assessment.

[46] Wagnild G. (2003). Resilience and Successful Aging: Comparison among Low and High Income Older Adults. J. Gerontol. Nurs..

[47] Ezeamama A.E., Elkins J., Simpson C., Smith S.L., Allegra J.C., Miles T.P. (2016). Indicators of resilience and healthcare outcomes: Findings from the 2010 health and retirement survey. Qual. Life Res..

[48] Martínez R.J., González L.P., Navarro N.E., De La Roca J.M., Reynoso O.U. (2021). Resilience Associated to Mental Health and Sociodemographic Factors in Mexican Nurses during COVID-19. Enferm. Glob..

[49] Vinaccia S., Quiceno J.M. (2011). Resiliencia y calidad de vida relacionada con la salud en pacientes con insuficiencia renal crónica—IRC. Rev. Argent. Clin. Psicol..

[50] Pidgeon A.M., Rowe N.F., Stapleton P., Magyar H.B., Lo B.C.Y. (2014). Examining Characteristics of Resilience among University Students: An International Study. Open J. Soc. Sci..

[51] Gómez D., Delgado U., Martínez F., Ortiz M., Áviles R. (2021). Resiliencia, género y rendimiento académico en jóvenes universitarios del Estado de Morelos. ConCiencia.

[52] Peñafiel-León J.E., Ramírez-Coronel A.A., Mesa-Cano I.C., Martínez-Suárez P.C. (2021). Impacto psicológico, resiliencia y afrontamiento del personal de salud durante la pandemia por COVID-19. Arch. Venez. Farmacol. Ter..

[53] Oducado R.M., Parreño-Lachica G., Rabacal J. (2021). Resiliencia personal y su influencia en el estrés, la ansiedad y el miedo de COVID-19 entre los estudiantes graduados en Filipinas. Int. J. Educ. Res. Innov..

[54] Gonçalves M.P., Freires L.A., Tavares J.E.T., Vilar R., Gouveia V.V. (2021). Fear of COVID and trait anxiety: Mediation of resilience in university students. Psicol.-Teor. Prática.

[55] Karataş Z., Tagay Ö. (2021). The relationships between resilience of the adults affected by the covid pandemic in turkey and COVID-19 fear, meaning in life, life satisfaction, intolerance of uncertainty and hope. Pers. Individ. Differ..

[56] Anyan F., Hjemdal O., Ernstsen L., Havnen A. (2020). Change in Physical Activity during the Coronavirus Disease 2019 Lockdown in Norway: The Buffering Effect of Resilience on Mental Health. Front. Psychol..

[57] Ozen B., Ceyhan O., Büyükcelik A. (2019). Hope and perspective on death in patients with cancer. Death Stud..

[58] Herth K. (1991). Development and refinement of an instrument to measure hope. Sch. Inq. Nurs. Pract. Int. J..

[59] Snyder C.R., Hoza B., Pelham J.W.E., Rapoff M., Ware L., Danovsky M., Highberger L., Ribinstein H., Stahl K.J. (1997). The development and validation of the Children’s Hope Scale. J. Pediatr. Psychol..

[60] Pereyra M.R. (2013). Test de Esperanza-Desesperanza: TED y TED-R.

[61] Cassaretto M., Martínez P. (2009). Validación de la Escala del Sentido de Humor en estudiantes universitarios. Rev. Psicol..

[62] Cowan T., Pham A.T., Elvevåg B., Cohen A.S. (2021). Social closeness and cognitive functioning increase feelings of hope for individuals in inpatient treatment. Psychiatry Res. Commun..

[63] Ripamonti C.I., Miccinesi G., Pessi M.A., Di Pede P., Ferrari M. (2016). Is it possible to encourage hope in non-advanced cancer patients? We must try. Ann. Oncol..

[64] Caycho Rodriguez T., Castilla Cabello H., Ventura Leon J.L. (2016). Esperanza en adolescentes y jóvenes peruanos: Diferencias según el sexo y la edad. Psychologia.

[65] Villacieros M., Bermejo J.C., Hassoun H. (2017). Validation of the scale of hope in terminal illness for relatives brief version (SHTI-b). validity and reliability analysis. An. Sist. Sanit. Navar..

[66] Gupta R., Mahajan R., Bakhshi A., Gupta K., Singh D., Kaur B. (2021). Fear vs. hope in India: Finding the silver lining amid the dark clouds of COVID-19. Pers. Individ. Differ..

[67] Limonero J.T., Tomás-Sábado J., Gómez-Romero M.J., Maté-Méndez J., Sinclair V.G., Wallston K.A., Gómez-Benito J. (2014). Evidence for validity of the brief resilient coping scale in a young spanish sample. Span. J. Psychol..

[68] MINSA Situación Actual “COVID-19” Perú—2020 (31 de Julio). https://www.dge.gob.pe/portal/docs/tools/coronavirus/coronavirus310720.pdf.

[69] Moreno C., Wykes T., Galderisi S., Nordentoft M., Crossley N., Jones N., Cannon M., Correll C.U., Byrne L., Carr S. (2020). How mental health care should change as a consequence of the COVID-19 pandemic. Lancet Psychiatry.

[70] Zapata-Ospina J.P., Patiño-Lugo D.F., Vélez C.M., Campos-Ortiz S., Madrid-Martínez P., Pemberthy-Quintero S., Pérez-Gutiérrez A.M., Ramírez-Pérez P.A., Vélez-Marín V.M. (2021). Intervenciones para la salud mental de estudiantes universitarios durante la pandemia por COVID-19: Una síntesis crítica de la literatura. Rev. Colomb. Psiquiatr..

[71] Aksoy A., Abic A., Degirmenci F., Vefikulucay D.Y. (2021). The relationship between quality of life and fear of Turkish individuals during the COVID-19 pandemic: A cross-sectional study. Arch. Psychiatr. Nurs..

[72] Servidio R., Bartolo M.G., Palermiti A.L., Costabile A. (2021). Fear of COVID-19, depression, anxiety, and their association with Internet addiction disorder in a sample of Italian students. J. Affect. Disord. Rep..

[73] Superio D.L., Anderson K.L., Oducado R.M.F., Luceño M.T., Palcullo V.E.V., Bendalian M.V.T. (2021). The information-seeking behavior and levels of knowledge, precaution, and fear of college students in Iloilo, Philippines amidst the COVID-19 pandemic. Int. J. Disaster Risk Reduct..

[74] Ahammed B., Jahan N., Seddeque A., Hossain T., Shovo T.-E., Khan B., Mamun M.A., Islam N. (2021). Exploring the association between mental health and subjective sleep quality during the COVID-19 pandemic among Bangladeshi university students. Heliyon.

[75] Villalba K.O., Avello R. (2019). Resilience as a factor determining satisfaction with life among university students. Rev. Cuba. Educ. Méd. Super..

[76] Vos L.M.W., Habibović M., Nyklíček I., Smeets T., Mertens G. (2021). Optimism, mindfulness, and resilience as potential protective factors for the mental health consequences of fear of the coronavirus. Psychiatry Res..

[77] Zheng D., Luo Q., Ritchie B.W. (2021). The Role of Trust in Mitigating Perceived Threat, Fear, and Travel Avoidance after a Pandemic Outbreak: A Multigroup Analysis. J. Travel Res..

[78] Araujo Hernández M., García Navarro S., García-Navarro E.B. (2021). Approaching grief and death in family members of patients with COVID-19: Narrative review. Enferm. Clin..

